# Upregulation of APOC1 Promotes Colorectal Cancer Progression and Serves as a Potential Therapeutic Target Based on Bioinformatics Analysis

**DOI:** 10.1155/2023/2611105

**Published:** 2023-03-01

**Authors:** Weiwei Tang, Hanyuan Liu, Xiao Li, Theng Choon Ooi, Nor Fadilah Rajab, Hongyong Cao, Razinah Sharif

**Affiliations:** ^1^Center for Healthy Ageing and Wellness, Faculty of Health Sciences, Universiti Kebangsaan Malaysia, Jalan Raja Muda Abdul Aziz, 50300 Kuala Lumpur, Malaysia; ^2^Hepatobiliary, Liver Transplantation Center, The First Affiliated Hospital of Nanjing Medical University, Key Laboratory of Living Donor Transplantation, Chinese Academy of Medical Sciences, Nanjing, Jiangsu, China; ^3^General Surgery, Nanjing First Hospital, Nanjing Medical University, Nanjing, Jiangsu, China; ^4^Biocompatibility Laboratory, Centre for Research and Instrumentation, University Kebangsaan Malaysia, 43600 UKM Bangi, Selangor Darul Ehsan, Malaysia

## Abstract

**Background:**

Approximately 10% of cancer patients worldwide have colorectal cancer (CRC), a prevalent gastrointestinal malignancy with substantial mortality and morbidity. The purpose of this work was to investigate the APOC1 gene's expression patterns in the CRC tumor microenvironment and, using the findings from bioinformatics, to assess the biological function of APOC1 in the development of CRC.

**Methods:**

The TCGA portal was employed in this investigation to find APOC1 expression in CRC. Its correlation with other genes and clinicopathological data was examined using the UALCAN database. To validate APOC1's cellular location, the Human Protein was employed. In order to forecast the relationship between APOC1 expression and prognosis in CRC patients, the Kaplan–Meier plotter database was used. TISIDB was also employed to evaluate the connection between immune responses and APOC1 expression in CRC. The interactions of APOC1 with other proteins were predicted using STRING. In order to understand the factors that contribute to liver metastasis from CRC, single-cell RNA sequencing (scRNA-seq) was done on one patient who had the disease. This procedure included sampling preoperative blood and the main colorectal cancer tissues, surrounding colorectal cancer normal tissues, liver metastatic cancer tissues, and normal liver tissues. Finally, an in vitro knockdown method was used to assess how APOC1 expression in tumor-associated macrophages (TAMs) affected CRC cancer cell growth and migration.

**Results:**

When compared to paracancerous tissues, APOC1 expression was considerably higher in CRC tissues. The clinicopathological stage and the prognosis of CRC patients had a positive correlation with APOC1 upregulation and a negative correlation, respectively. APOC1 proteins are mostly found in cell cytosols where they may interact with APOE, RAB42, and TREM2. APOC1 was also discovered to have a substantial relationship with immunoinhibitors (CD274, IDO1, and IL10) and immunostimulators (PVR, CD86, and ICOS). According to the results of scRNA-seq, we found that TAMs of CRC tissues had considerably more APOC1 than other cell groups. The proliferation and migration of CRC cells were impeded in vitro by APOC1 knockdown in TAMs.

**Conclusion:**

Based on scRNA-seq research, the current study shows that APOC1 was overexpressed in TAMs from CRC tissues. By inhibiting APOC1 in TAMs, CRC progression was reduced in vitro, offering a new tactic and giving CRC patients fresh hope.

## 1. Introduction

Having a high rate of morbidity and death, colorectal cancer (CRC) is a malignant tumor of the digestive system [1]. Around 10% of all cancer patients globally have CRC, affecting 1.36 million persons [2]. The liver is the most common site of hepatic metastasis in CRC because of its close anatomical proximity. At the time of initial diagnosis, 20–25% of patients had CRC liver metastases at some point, later on, 50–60% will [3]. Currently, hepatectomy, which has a 60% 5-year survival rate, is the best course of treatment for CRC patients with liver metastases. Unfortunately, approximately 20–25% of patients with CRC liver metastases are eligible for resection at the time of diagnosis, leading to the majority of patients eventually passing away from advanced metastases [4].

The influence of immunology on the development, prognosis, and therapeutic response of CRC has been shown in recent investigations. A better prognosis, for instance, is linked to *T* and NK cell enrichment in original CRC or metastases [5, 6]. Programmed cell death 1 (PD1), T cell control of CD28 superfamily members, and PD ligand 1 (PD-L1) are examples of immune checkpoint molecules that have recently been identified as potential targets for CRC immunotherapy [7]. Pembrolizumab (Keytruda®) was the first PD1 inhibitor authorized by the FDA for the treatment of metastatic malignant melanoma [8]. The historic clinical trial of pembrolizumab for CRC, NCT01876511, is noteworthy. The clinical trial included 11 patients with deficient mismatch repair gene expression (dMMR) colorectal cancer, 21 patients with proficient mismatch repair gene expression (pMMR) colorectal cancer, and 9 patients with dMMR other malignancies. In dMMR CRC patients and pMMR CRC patients, respectively, the immune-related objective response rate and the immune-relatedprogression-free survival (PFS) rate were 40% and 78% and 0% and 11%. While the pMMR CRC group's median PFS and overall survival (OS) were 2.2 months and 5.0 months, respectively, the dMMR group did not accomplish these milestones [9]. Patients with CRC now have a lot of optimism because of PD1 monoclonal antibody therapy, but medication resistance is still a concern that needs to be fixed. The study of the molecular mechanisms underlying the CRC tumor microenvironment is so crucial.

In the current work, we employed bioinformatics technology to evaluate the TCGA database and discovered that apolipoprotein C1 (APOC1) expression was considerably higher expressed in CRC tissues than in nearby tissues and that it was associated with clinical stage and a bad prognosis. The selection of APOC1 to study its role in the CRC tumor microenvironment was inspired by our research group [10]. Although APOC1 has been observed to be crucial for the growth and metastasis of a number of malignancies [11], the underlying mechanisms have not been fully understood, particularly with regard to its function and part in tumor immunity [12]. Liwen Ren et al. established that APOC1 is an immunological biomarker that controls macrophage polarization and encourages tumor dissemination through extensive pan-cancer studies, which revealed that APOC1 is intimately connected to the infiltration of different immune cells in a range of malignancies [13]. We conducted single-cell RNA sequencing (scRNA-seq) in one patient with CRC liver metastasis to further examine why APOC1 is highly expressed in CRC tissues. We covered primary colorectal cancer tissues (CT), neighboring colorectal cancer tissues (CP), liver metastatic cancer tissues (LT), normal liver tissues (LP), and preoperative blood (PB) in order to determine which type of cell population APOC1 is significantly enriched for and address causes of liver metastasis from CRC. Furthermore, we conducted a preliminary evaluation of APOC1's role in encouraging CRC migration and proliferation in culture.

## 2. Materials and Methods

### 2.1. APOC1 Expression Level Analysis and Clinicopathological Analysis in CRC

The expression of APOC1 was examined in 24 different tumor tissue types, including CRC and related para-carcinoma tissues, using the TCGA portal. Here, UALCAN was utilized to compare the expression of APOC1 in CRC patients with various stages and lymph node metastases [14, 15].

### 2.2. Tools for APOC1 Location in Cells

A large number of tissue, cellular, and pathological results as well as gene data in cells and tissues are compiled in the Human Protein Atlas, through which we obtained the location of APOC1 in cells. The subcellular portion of the Human Protein provides high-resolution insight into the expression and spatial and temporal distribution of proteins encoded by 13,041 genes, representing 65% of human protein-coding genes. For each gene, the subcellular distribution of proteins was studied by immunofluorescence (ICC-IF) and confocal microscopy in up to three different cell lines selected from the 36 cell lines found in cell line sections. We showed the colocalization of APOC1 in three cell lines including A-431, U-2 OS, and U-251 MG cell lines.

### 2.3. Interaction Analysis of APOC1

STRING is a public database for searching for interactions and connections between proteins, both direct and indirect [16]. We perform a thorough analysis and forecast of the outcomes, as well as a summary of information exchange and contact with other (primary) databases. STRING was used to build a network of connections between APOC1, APOE, RAB42, and TREM2 among other significant proteins. The relationship between APOC1 and other genes in CRC was examined using the TCGA portal.

### 2.4. scRNA-Seq Analysis

In accordance with the principles outlined in the Declaration of Helsinki, all participants were given information about the study, and patients signed informed consent was obtained before it could begin. After surgical resection at the First Affiliated Hospital of Nanjing Medical University, primary colorectal cancer tissues and corresponding intestine tissues, liver metastatic cancer tissues, and corresponding liver tissues were collected. Additionally, preoperative blood was taken. An Illumina Hiseq4000 sequencer evaluated the samples (Singleron Biotechnologies, China). Using the 10X Genomics CellRanger workflow, the raw counts were compared to the human reference provided by 10X Genomics (GRCh38 version) (version 2.1.0). The filter expression matrix CellRanger generates for each sample are read and processed using the R program Seurat. Additionally, Seurat was used to checking the quality of single-cell expression matrices (version 3.2.0). First, cells that met the following standards for quality were eliminated: mitochondrial transcripts with less than 15% uniqueness, less than 100 unique genes mapped, and more than 500 unique molecular identification counts (UMI). Using the default settings of the R program DoubletFinder, double peaks in cells were found. The leftover cells in all samples were kept and subsequently merged with Seurat for additional analysis, assuming that the duplex was eliminated.

### 2.5. Cell Cluster Analysis and Cell Type Identification

Using FindVariable features from Seurat, 2000 highly variable genes (HVG) were generated, and these genes were employed in principal component analysis (PCA) with parameter NPCS = 30. The Harmony program (version 0.1.0) was used to eliminate probable batches from samples with the parameter NPCA = 12 based on the PCA results. FindClusters from Seurat are then used to identify cell clusters using a shared nearest-neighbor graph. Seurat's RunTSNE and RunUMAP are used to reduce the harmony dimension of visualization by T-distributed random neighborhood embedding (tSNE) and unified manifold approximation and projection (UMAP).

### 2.6. Tool for Immune-Related Analysis of APOC1

The spearman correlations between APOC1 and immune-modulator expression were investigated using TISIDB, a digital portal for tumor and immune system interactions that integrates a variety of heterogeneous data [16].

### 2.7. Primary Culture of THP-1 and Cell Transfection

10% fetal bovine serum (FBS) was added to RPMI 1640 medium (BI, USA) when cultivating THP-1 cells (Gibco, USA), following the transfection of THP-1 cells with lentiviral vectors, including sh-NC and sh-APOC1 (GeneChem, China). sh-NC:5′-TTCTCCGAACGTGTCACGT-3′; sh-APOC1:5′-GCATCAAACAGAGTGAACTTT-3′. After two days of PMA-mediated macrophage differentiation, THP-1 cells were collected. THP-1 cells were exposed to HCT116/LOVO culture supernatant in RPMI 1640 media for 2 days in tumor-associated macrophages (TAMs) stimulation tests, which resulted in the production of TAMs.

### 2.8. Cell Proliferation Experiments

For CCK8 experiments, CRC cells were cocultured with TAMs supernatant containing sh-NC or sh-APOC1. Ten microliters of CCK8 solution (RiboBio, China) were applied at 0 hours, 24 hours, 48 hours, and 72 hours after the cocultured tumor cells were implanted in 96 wells. 4 hours after adding the CCK8 solution, analyses were carried out using a microplate reading element at 450 nm in accordance with the manufacturer's instructions (Synergy4, USA).

### 2.9. Scratch Wound Experiment

At 48 h after transfection, after cells were adherent into monolayer cells, the scratched cells were evenly crossed with a sterile gun tip, gently washed with PBS, and then replaced with 1% FBS medium and cultured in a 37°C and 5% CO_2_ incubator. At 0 h and 48 h, 5 fields were randomly selected under an inverted microscope to observe the wound healing and take photos. Cell migration distance was measured and calculated.

### 2.10. Statistics-Related Analyzing Process

Continuous information was compared between the two groups by one individual *t*-test procedure. GraphPad Prism 8.0 was used to carry out the statistically significant analytical method and present the results graphically. It was deemed statistically significant with a *P* value of 0.05.

## 3. Results

### 3.1. Expression and of Clinical Role of APOC1 in CRC Based on TCGA Data

**Figure 1:**
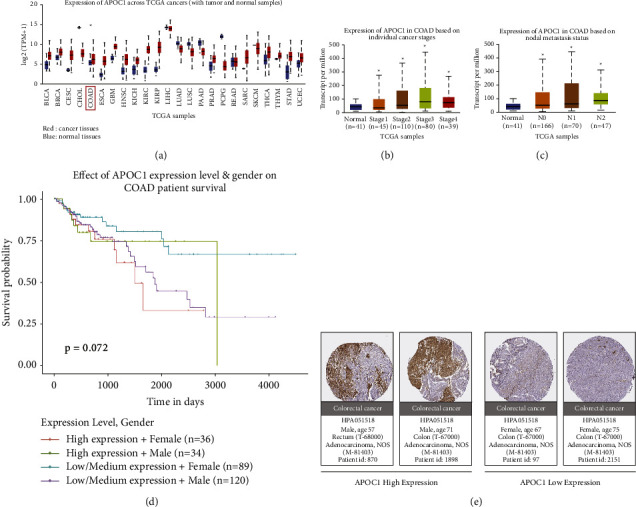
Expression of APOC1 in CRC tissues. (a) The expression level of APOC1 mRNA in different types of cancer tissues compared to normal tissue. (b) The correlation between APOC1 mRNA expression and tumor stage. (c) The correlation between APOC1 mRNA expression and lymph node metastatic status. (d) The relationship between APOC1 expression and CRC patient's prognosis. (e) Immunohistochemical of APOC1 expression in CRC tissues from different patients. ^*∗*^*P* < 0.05.

The TCGA portal revealed that the expression of APOC1 was higher in tumor tissues, including CRC, than in normal tissues (Figure 1(a)). Based on subgroup analysis of CRC individual cancer stages and lymph node metastasis, it was discovered that APOC1 expression increased with increasing cancer stage and lymph node metastasis (Figures 1(b) and 1(c)). Using the Kaplan–Meier plotter, the prognostic value of APOC1 in CRC was further investigated. The findings revealed that, albeit not statistically significant (*p* > 0.05), CRC patients with high APOC1 expression had considerably worse prognoses than those with low expression (Figure 1(d)). Data from the Human Protein Atlas analysis showed that CRC patients, including those with rectum and colon cancer, had high or low expression of the APOC1 protein (Figure 1(e)).

### 3.2. Genes and Proteins Cointeracted with APOC1 in CRC

**Figure 2:**
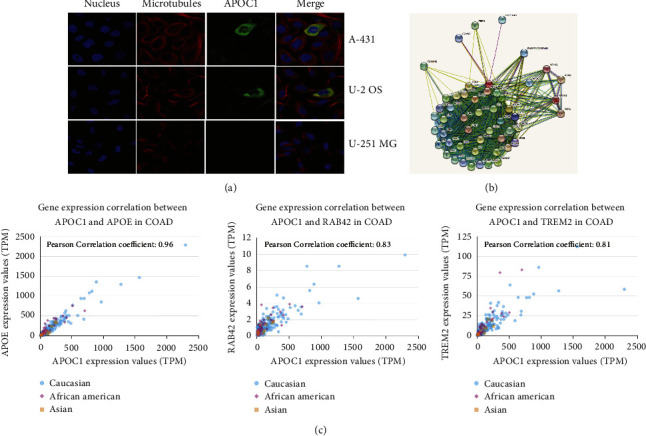
Genes and proteins cointeracted with APOC1. (a) APOC1 located in the cytosol. (b) Interactions between APOC1 and other proteins. (c) Relationship analysis between APOC1 and APOE, RAB42, and TREM2 in CRC.

The human protein Atlas database revealed that APOC1 was found in the cytoplasm of A-431, U-2 OS, and U-251 MG cells (Figure 2(a)). It was possible to find proteins that interact with APOC1 using a STRING interactive network (Figure 2(b)). Further investigation revealed a high correlation between the expression of APOC1 and proteins that may interact with it, including APOE, RAB42, and TREM2 (Figure 2(c)).

### 3.3. Acquisition of scRNA-Seq Profiles of Samples and Data Generation in CRC Liver Metastasis

**Figure 3:**
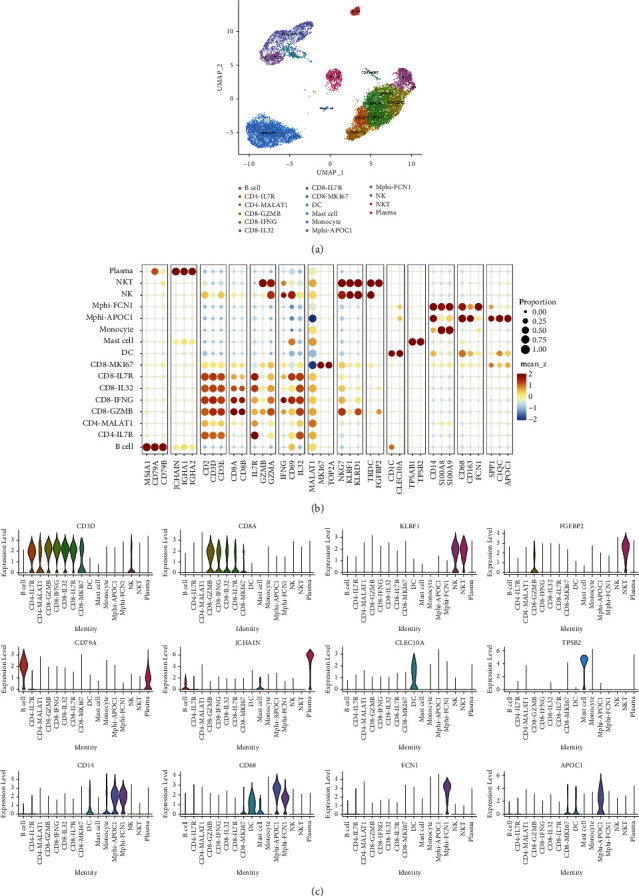
Diverse cell types in CRC delineated by single-cell transcriptomic analysis. (a) UMAP plot showing 16 clusters of immune cells. (b) Dot plot showing the clustering of immune cell types in each sample. (c) The violin diagram showing expression levels of specific markers in each cell type.

**Figure 4:**
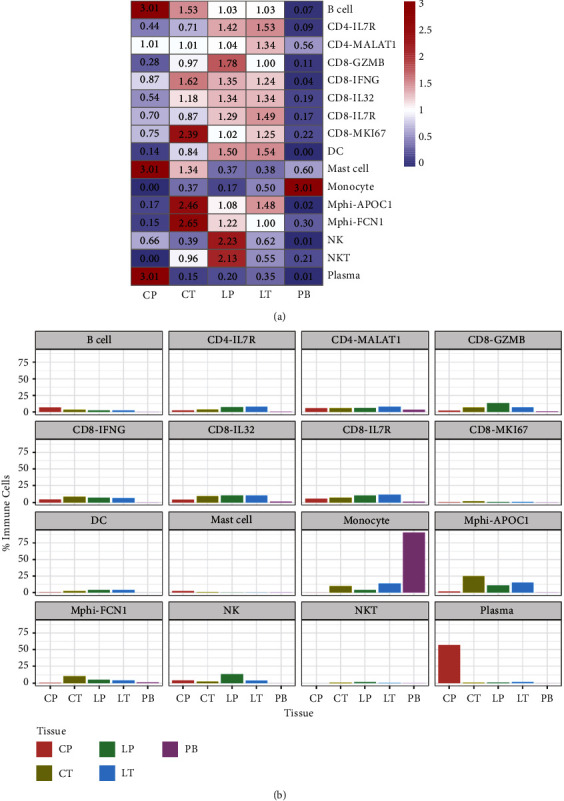
Expression of individual cell populations in individual samples. (a) The distribution number of each immune cell subgroup in each sample. (b) The histogram showing the distribution number of each immune cell subgroup in each sample. CT, primary colorectal cancer tissues; CP, adjacent tissues of colorectal cancer; LT, liver metastatic cancer tissues; LP, normal liver tissues; PB, preoperative blood.

In this study, we carried out scRNA-seq [17] in one CRC liver metastasis patient covering primary colorectal cancer tissues (CT), adjacent tissues of colorectal cancer (CP), liver metastatic cancer tissues (LT), normal liver tissues (LP), and preoperative blood (PB) and aimed to address the causes of liver metastasis from CRC. Through the definition of classification, we finally identified 16 cell clusters in immune cells using a UMAP plot (Figure 3(a)). Each cell type has unique maker genes (Figure 3(b)). For example, the B cell cluster specifically expresses MS4A1, CD79A, and CD79B. CD4-IL7R expresses IL7R, IL32, and MALAT1; while CD8-GZMB expresses CD8A, CD8B, and KLRD1. TAM-APOC1 expresses CD14, C1QC, APOC1, and SPP1. In addition, we used the violin chart to show the expression of some marker genes (CD79A, FCN1, and APOC1) in various cell populations (Figure 3(c)). A cluster map and histogram were applied to show the expression of these cell clusters in different tissues and results revealed that there was less TAM but more B and plasma cell clusters in CP compared with CT. CT showed more TAM and fewer CD8 T cells. Moreover, there were more CD8 T cells and NK enrichment in LP compared with LT (Figures 4(a) and 4(b)). These results demonstrate that TAMs might play an important role in both the metastasis of primary tissues and the colonization of metastatic foci and that the lethality of NK cells in cancer tissues is insufficient in the colonization process after metastasis of CRC.

### 3.4. APOC1 Was Highly Expressed in TAMs of CRC Tissues

**Figure 5:**
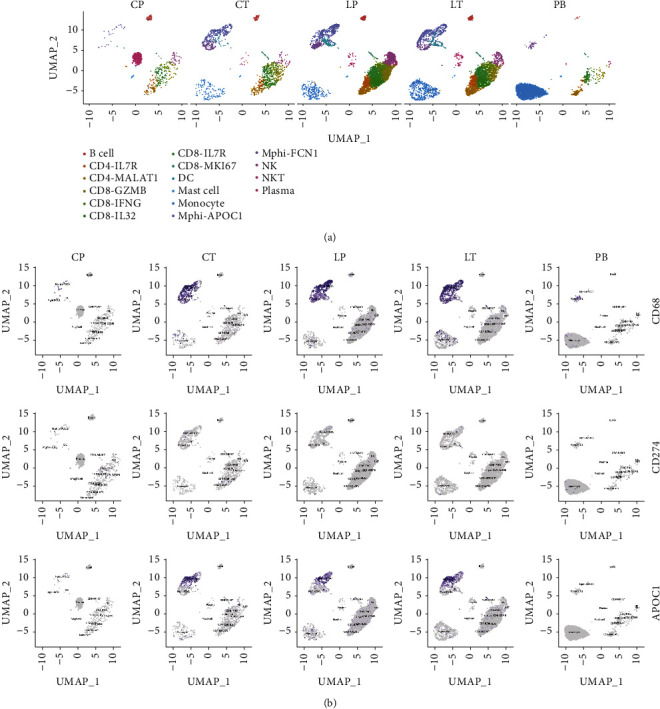
APOC1 expression in individual cell populations and individual samples. (a) UMAP plot showing the distribution of each cell subgroup in each sample. (b) The expression of APC1 in immune cells in each sample. CT, primary colorectal cancer tissues; CP, adjacent tissues of colorectal cancer; LT, liver metastatic cancer tissues; LP, normal liver tissues; PB, preoperative blood (PB).

**Figure 6:**
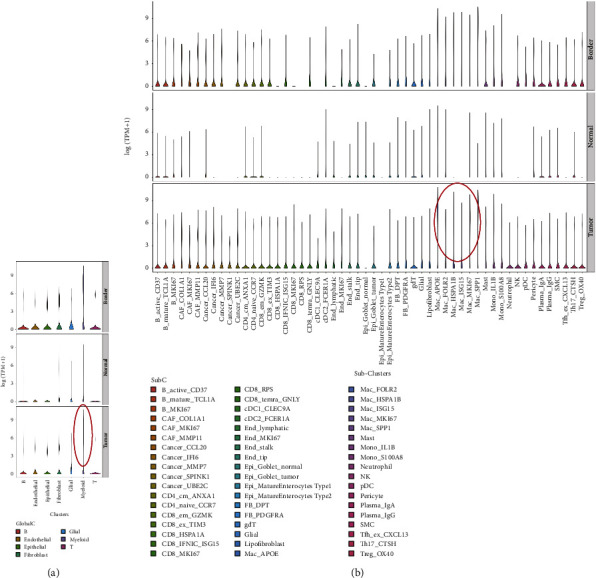
Research results of APOC1 at single-cell level from Hae-Ock Lee's study. (a) The violin diagram displaying the distribution of APOC1 expression in different cells from CRC tissues in total analysis. (b) The violin diagram displaying the distribution of APOC1 expression in different cells from CRC tissues in subanalysis.

We discovered that APOC1 may be crucial to the TAMs of CRC based on the results of the scRNA-seq analysis. As a result, we carefully examined the expression of APOC1 in each sample and each cluster of cells. The exact distribution of various subgroups in various samples is also displayed in the UMAP graphic (Figure 5(a)). The enrichment of APOC1 in various cell clusters in various samples was more clearly displayed by the UMAP map (Figure 5(b)). TAMs had much higher levels of APOC1 than other cell clusters such as CD8 T, CD4 T, and NK cells, which were both less expressed (Figure 5(b)). Furthermore, we looked at the data from Hae-Ock Lee et al. [18] and discovered that APOC1 was primarily expressed in myeloid CRC tissues (Figures 6(a) and 6(b)), which is similar to our findings. Our intense curiosity about the function of APOC1 in TAMs from CRC was piqued by all of these analyses.

### 3.5. APOC1 Expression Was Correlated with Immune Factors

**Figure 7:**
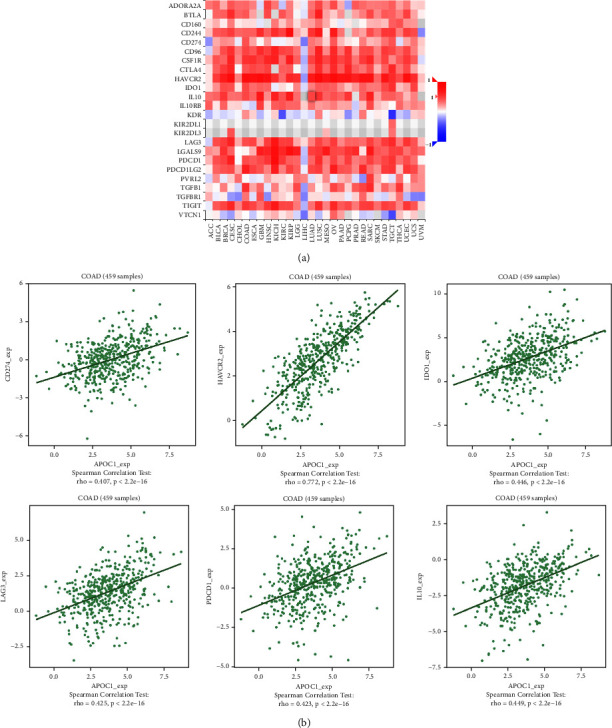
Correlation between APOC1 expression and immunoinhibitors in CRC. (a) The heat map showing the correlation between APOC1 and immunoinhibitor factors in different cancers. (b) The line graph showing the correlation of APOC1 with specific immune indicators in CRC.

**Figure 8:**
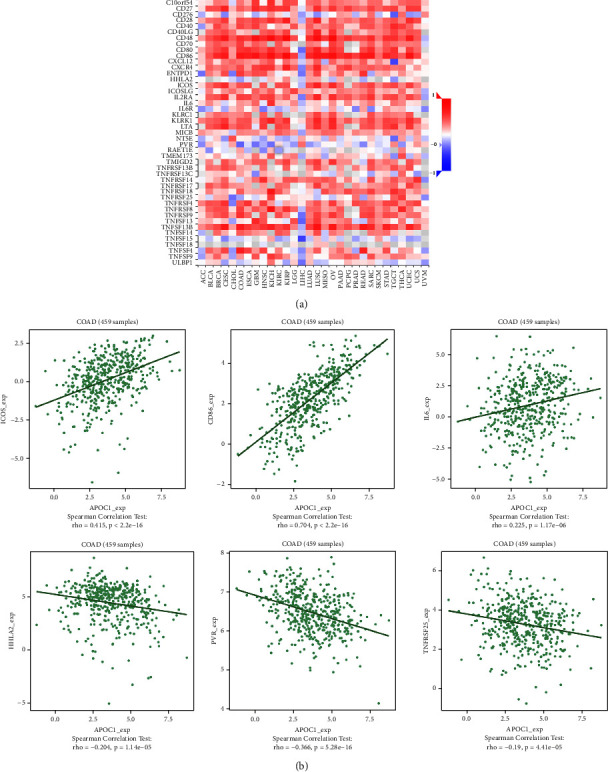
Correlation between APOC1 expression and immunostimulators in CRC. (a) The heat map showing the correlation between APOC1 and immunostimulator factors in different cancers. (b) The line graph showing the correlation of APOC1 with specific immune indicators in CRC.

We investigated the connection between the expression of APOC1 and immunological components in CRC because the aforementioned findings showed that APOC1 is linked to immunity, particularly TAMs, in CRC. As shown in Figures 7 and 8, there was a significant link between the expression of immunostimulators (PVR, CD86, and ICOS) and immunoinhibitors (CD274, IDO1, and IL10) and APOC1 itself.

### 3.6. Inhibition of APOC1 of TAM Reduced CRC Progression In Vitro

**Figure 9:**
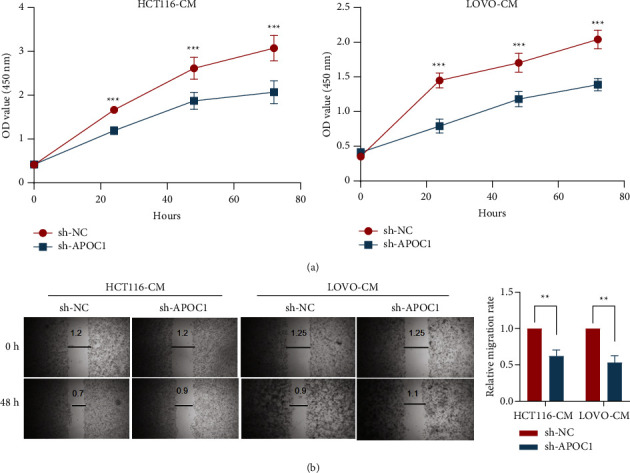
Inhibition of APOC1 of TAMs reduced CRC progression in vitro. (a) CCK8 assay of CRC cancer cells with TAM supernatant in different groups. (b) Scratch assay of cancer cells with TAM supernatant in different groups. ^*∗∗*^*P* < 0.01, ^*∗∗∗*^*p* < 0.001.

We stimulated CRC cells with TAM supernatant in order to further confirm the function of APOC1 in TAMs from CRC in vitro. By using the CCK8 and scratch assays, we discovered that TAMs in the sh-APOC1 group significantly decreased the proliferation and migration of CRC cells in comparison to the control group (Figures 9(a) and 9(b)).

## 4. Discussion

An earlier study found that the mitogenic impact of high-density lipoprotein cholesterol (HDL) on bovine vascular endothelial cells in vitro was caused by APOC1 purified from HDL [19], and APOC1 has recently been identified as a molecule involved in the advancement of cancer. According to research, APOC1 functions as an oncogene in cervical cancer, and its knockdown both in vitro and in vivo reduces the proliferation of cervical cancer cells. The clinical outcome of cervical cancer patients is highly correlated with the relative expression of APOC1 [20]. Li Yangling et al. discovered that APOC1 activated STAT3 to increase renal clear cell carcinoma metastasis [11]. According to Huaying Xiao et al., clear cell renal cell carcinoma (ccRCC) tissues had a greater expression level of APOC1 than the normal group did. Poor prognosis was linked to high APOC1 expression in female patients but not in male patients. In ccRCC patients older than 60 years, high APOC1 expression also decreased survival [21]. Through the MAPK signaling pathway, APOC1 increases the growth of CRC tumors, according to research by Ren Hui et al. [12]. Based on the TCGA portal, we discovered in the current study that the expression of APOC1 in tumor tissues, including CRC, was obviously higher than that in normal tissues. Subgroup analysis also revealed that the higher the cancer stage and lymph node metastasis, the higher the expression of APOC1. Results from the Kaplan–Meier plotter demonstrated that, despite being not statistically significant, the prognosis of CRC patients with high APOC1 expression was significantly worse than that of those with low expression. Our conclusion is generally in line with the previous conclusion.

A STRING interactive network revealed a favorable correlation between the expression of APOC1 and proteins like APOE, RAB42, and TREM2 that may interact with APOC1. Both APOC1 and APOE are apolipoproteins, which function as physiological carriers of hydrophobic lipids in aqueous fluids throughout the body [22]. Apolipoproteins and different malignancies may be related, according to some research studies. In lung cancer cells and B16F10 cells, APOE expression was knocked down, which reduced tumor development and metastasis [23]. To describe APOE-TREM2 interactions, molecular docking and molecular dynamics (MD) investigations were carried out. Additionally, it was examined how a significant TREM2 disease-related mutation (R47H) affected TREM2 affinity for APOE. The outcomes demonstrated that the binding energy occurred between APOE and TREM2 in an isomer-dependent manner, with the potency order being APOE4 > APOE3 > APOE2. Furthermore, the R47H mutation decreased the connection between the APOE and TREM2 proteins, which may be a result of hydrogen bond interactions, hydrophobic interactions, or a weaker electrostatic interaction between APOE and TREM2 [24]. RAB42 is linked to cancer prognosis and progression, according to earlier studies. RAB42 expression levels in hepatocellular carcinoma (HCC) tissues were higher than in normal tissues, according to a prior investigation. Significant correlations were found between highly expressed RAB42 and a number of clinical indicators in HCC patients. Additionally, elevated RAB42 expression unmistakably indicated a bad prognosis for HCC [25]. In comparison to normal samples, glioblastoma (GBM) samples showed higher expression of RAB42. Patients with high RAB42 expression have a worse prognosis than those with low RAB42 expression in GBM. A total of 35 pathways, including the P53 pathway, were significantly activated in GBM samples with elevated RAB42 expression [26]. A greater understanding of the direct interactions between APOC1, APOE, RAB42, and TREM2 in cancer is, however, required due to the paucity of studies in this area.

The role and mechanism of APOC1 in the tumor microenvironment have only been partially studied. With the quick advancement of scRNA-seq technology, diverse cell populations can be characterized and identified, and new cell markers and regulatory pathways can be found. It is interesting to note that APOC1 has been linked to a number of immune cell infiltrations in different malignancies. scRNA-seq research revealed that TAMs expressed the bulk of APOC1 in expression. TAMs with the M2 phenotype are produced when renal cell cancer cells are cocultured; this is prevented by silencing APOC1. By interacting with CD163 and CD206, APOC1 boosted macrophage polarization toward M2 by increasing its expression in M2 or TAM. Additionally, through secreting CCL5, macrophages overexpressing APOC1 aided in the spread of renal cell cancer cells [13]. According to Chan et al.'s research, TAMs have high levels of APOE, APOC1, and SPP1 expression, which results in an anti-inflammatory macrophage phenotype [27]. Based on the findings of this study's scRNA-seq, we show that basic CRC and liver metastatic tissues exhibit APOC1 overexpression in TAMs. We also looked at the relationship between the expression of APOC1 and immune factors in CRC and discovered that there was a significant positive correlation between the expression of immunoinhibitors (CD274, IDO1, and IL10) and the expression of APOC1. This result suggested that APOC1 is important in the development of the immunosuppressive tumor microenvironment. We stimulated CRC cancer cells with TAM supernatant in order to further confirm the function of APOC1 in CRC TAMs in vitro. TAMs in the sh-APOC1 group drastically decreased CRC cell proliferation and migration by CCK8 and scratch assays. The significance of APOC1 in the tumor immune microenvironment is substantially expanded by our findings. Massimo Pancione et al. proposed that many different functions of TAMs during tumor progression may depend on their intrinsic adaptation to positional schemes that are acquired through factors that control the balance between a tumor suppressor and tumor-promoting activities. In the primary tumor, oncogenic alterations or changes in the tumor microenvironment establish a new equilibrium that can be further modified during metastasis. There are at least two mechanisms supporting the prometastatic function of TAMs: (1) M2-macrophages can form a dense barrier around invasive cancer cells, leading to heterotypic interactions between tumor cells and the surrounding matrix, disrupting host tissue integrity; (2) Invasive cancer cells can acquire immunophenotypic features, such as macrophage fusion with cancer cells, which promotes homotypic interactions between host matrix and TAMs [28–30]. Therefore, TAMs play an indispensable role in the progression of liver metastasis of CRC.

The association between APOC1 and CRC was extensively investigated in this research using bioinformatics analysis and a few trials, although there are still numerous gaps in our understanding. First off, no mechanistic investigation was done; only APOC1's expression and function in TAMs were confirmed. Second, there are not many research studies on in vivo efficacy in animals. Third, the impact of APOC1 knockdown on other cells was not investigated in TAMs. We anticipate publishing more information about the connection between APOC1 function and cancer in many cell types.

## 5. Conclusion

In conclusion, the current study shows that APOC1 was highly expressed in TAMs of CRC tissues based on scRNA-seq and bioinformatics analysis and that inhibiting APOC1 of TAMs slowed CRC progression in vitro, offering a novel approach and giving CRC patients fresh hope.

## Data Availability

All data relevant to the study are included in the article.
